# Editorial: Biomarkers and immunotherapy of hepatic-biliary-pancreatic cancers

**DOI:** 10.3389/fonc.2023.1301416

**Published:** 2023-10-23

**Authors:** Yawei Qian, Wenyu Jia, Hongda Liu

**Affiliations:** ^1^ Department of General Surgery, The First Affiliated Hospital of Nanjing Medical University, Nanjing, China; ^2^ Department of Endocrinology, Qingdao Municipal Hospital, Qingdao, Shandong, China

**Keywords:** hepatic-biliary-pancreatic cancers, biomarker, therapeutic strategy, drug resistance and metastasis, clinical studies

## 1 Introduction

Hepatic-Biliary-Pancreatic (HBP) cancers represent a formidable challenge within the broader spectrum of gastrointestinal malignancies (1). These cancers encompass a complex array of diseases affecting the liver, bile ducts, and pancreas. Incidence rates for HBP cancers have been on the rise, a concerning trend attributed to various factors, including changes in dietary patterns and exposure to environmental pollutants (2). The treatment landscape for HBP cancers, much like other gastrointestinal malignancies, comprises a multifaceted approach that encompasses surgical interventions, chemotherapy, radiotherapy, and the more recent addition of immunotherapy (3–5).

Despite substantial strides in cancer research and treatment modalities, HBP cancers continue to pose significant hurdles. These challenges manifest as recurrent tumors, metastasis to distant organs, and the development of drug resistance, all of which can thwart the prospects of complete recovery (6). The burgeoning field of oncology is witnessing a transformation fueled by innovative research technologies. Genomics, high-throughput sequencing, proteomics, metabolomics, immunotherapy, nanotechnology, liquid biopsy, robotic surgery, artificial intelligence, organoids, and microbiome analysis are among the cutting-edge tools being rapidly integrated into clinical and biomedical research.

These avant-garde approaches have yielded a treasure trove of information that is reshaping our comprehension of HBP cancers. The insights gleaned from these novel techniques have cast new illumination on existing theories, prompting a reevaluation of established paradigms and doctrines. Such breakthroughs have the potential to chart novel pathways of understanding, ultimately leading to more precise and efficacious treatments with fewer adverse events for patients. By keeping abreast of the latest research findings, clinicians and researchers can identify emerging diagnostic and prognostic factors, biomarkers, and risk factors that hold the promise of improving our grasp of the molecular underpinnings of cancer initiation, progression, recurrence, and drug resistance (7).

Furthermore, the exploration of targeted anti-cancer agents represents a pivotal avenue for enhancing the traditional armamentarium of chemotherapy and radiotherapy in combating HBP cancers (8). These efforts are pivotal in advancing the field and offer renewed hope for patients grappling with these formidable diseases. As reported in the Research Topic of Biomarkers and Immunotherapy of Hepatic-Biliary-Pancreatic Cancers, we can anticipate significant strides in the diagnosis, prognosis, and ultimately the survival of patients affected by these intricate malignancies. In sum, the confluence of cutting-edge research methodologies and unwavering dedication to improving patient outcomes promises a brighter future in the fight against Hepatic-Biliary-Pancreatic cancers.

## 2 Hepatocellular carcinoma

HCC, also known as primary liver cancer, is a formidable malignancy originating from hepatocytes, the primary functional cells within the liver. Its incidence has seen a steady rise in recent decades, contributing to its status as a significant global health concern (9). HCC is predominantly associated with chronic liver diseases, including hepatitis B and C infections, excessive alcohol consumption, non-alcoholic fatty liver disease (NAFLD), and metabolic disorders, particularly cirrhosis (10). Interestingly, in patients with Budd-Chiari syndrome (BCS), the risk factors for developing HCC were examined by (Li K. et al.). Among 113 BCS patients studied, 10.6% (12/113) were diagnosed with HCC. Those who had BCS-associated HCC tended to be older and had elevated serum AST and total bilirubin levels. HCC nodules in these patients were typically found in the right posterior lobe and showed irregular and heterogeneous enhancement during the arterial phase with washout during the delayed phase on CT imaging. The study suggests that BCS patients with IVC block and hepatic venous outflow tract stricture may be at higher risk for HCC development.

The aggressive nature of HCC, coupled with its often late-stage diagnosis, has limited therapeutic options, making it a formidable challenge for healthcare professionals. However, recent advancements in genomics and molecular biology have illuminated the underlying mechanisms of HCC and hold the promise of improved patient outcomes. Zhu Z. et al. reported that high expression of polycomb repressive complex 2 component, PHF19, was correlated with poor prognosis of HCC. PHF19 expression related to tumor mutational burden and immune infiltrates, notably myeloid-derived suppressor cells and Th2 CD4+ T cells. Enrichment analyses linked PHF19 to cell cycle, DNA replication, and immune processes, thus highlighting PHF19 as an epigenetic regulator affecting cancer progression and immune infiltration, with potential clinical implications. Huang et al. investigated the role of Actin-related protein 2/3 complex subunit 5 (ARPC5) in various cancers. ARPC5 was found to be upregulated in most cancer types and associated with worse prognosis in certain cancers. It exhibited low tissue and cell specificity in normal tissues and was linked to tumor microenvironment scores, immune cell infiltration, and immune-related genes in many cancers. Additionally, ARPC5 was positively correlated with factors like TMB, MSI, and RNA modification genes in specific cancers. In their experimental analyses, ARPC5 was found to promote proliferation, migration, and invasion in HCC. Yu et al. examined the role of Complement Factor H-related 4 (CFHR4) in HCC, which was significantly reduced in HCC tissues and was associated with various clinicopathological factors. Functional analysis revealed its potential involvement in several biological pathways, including carcinogenesis and metabolic pathways. CFHR4 expression correlated with immune cell infiltration, affecting various immune cell types. High CFHR4 expression was linked to better survival outcomes in HCC patients. The study also constructed potential CFHR4-related regulatory networks. Xiang et al. found that spermine synthase (SMS), involved in polyamine biosynthesis, is overexpressed in HCC. SMS overexpression in HCC patients is unrelated to hepatitis virus infection and is associated with poor prognosis. High SMS levels are linked to decreased survival rates and limited effectiveness of immune checkpoint blockade (ICB), therefore may impact HCC development by affecting various immune-related pathways. Liu Y. et al. investigated the role of CEP192 in HCC and found that its expression increased with tumor stage and was linked to poor clinical features, recurrence, and higher mortality. CEP192 played a role in the proliferation and self-renewal of hepatic progenitor-like cells, and silencing it inhibited cell proliferation. CEP192 was also associated with immunosuppressive elements in the tumor microenvironment, suggesting it could predict responses to immune checkpoint inhibitors. Cai L. et al. reported that transmembrane protein 88 (TMEM88) plays a role in the canonical Wnt signaling pathway. Their study analyzed TMEM88 expression in HCC, revealing a negative correlation with tumor stage and grade. High TMEM88 levels predicted better overall and disease-specific survival. TMEM88 overexpression reduced HCC cell proliferation *in vitro* and suppressed HCC progression in a mouse model. Interestingly, Ai et al. developed a bioinformatics pipeline that accurately estimates the number of infiltrating immune cells and bacteria in tumor and normal tissues. Using this pipeline on liver cancer samples, they identified specific bacteria and immune cell types that differ between healthy and diseased tissue, achieving an 84% accuracy in distinguishing them. This tool can help researchers better understand the interactions between immune cells, bacteria, and cancer cells.

In addition to single prognostic biomarkers, a large proportion of studies are now focusing on establishing prognostic model using multiple molecules. Zhang H. et al. identified four distinct tumor microenvironment (TME) subtypes (C1, C2, C3, C4) based on immune, stem, and stromal cell compositions. C1 and C2 exhibited an immune-active TME, while C3 and C4 displayed an immune-insensitive TME. Patients in the C3 subtype had notably worse prognoses, demonstrating the potential for personalized treatment approaches in HCC based on TME subtypes. Liver zonation, characterized by distinct functions across the radial axis of the liver lobule, can impact the development of liver cancer, as reported by (Zhang T. et al.). Their study identified hepatocyte-specific zonation markers and used them to classify HCC into three clusters: non-zonational-like, central-like, and portal-like. Each cluster exhibited different clinical characteristics, immune infiltration, and prognosis. Chen J. et al. identified 16 differentially expressed genes (DEGs) related to liver cancer immunotherapy using machine learning. A CombinedScore based on these DEGs predicted patient response to immunotherapy. Patients with a low CombinedScore were likely to respond better. Metabolism pathways were more active in patients with a high CombinedScore. The CombinedScore was associated with immune cell levels, immune checkpoint expression, and genomic features. Specifically, CDCA7 was identified as a potential therapeutic target and was linked to macrophage polarization and T cell activity. Sun et al. identified two major subtypes of HCC based on the expression of Golgi apparatus-related genes (GARGs). The high-risk subtype (C1) had lower survival rates and poorer response to immunotherapy, along with characteristics indicating immune escape and TP53 mutations. A risk assessment profile based on GARGs was developed, helping predict prognosis and immunotherapy response in HCC patients. Additionally, the study found that interfering with the expression of the BSG gene restricted the proliferation and migration of HCC cells, suggesting it may be associated with poor HCC prognosis. Luo et al. focused on the role of cancer-associated fibroblasts (CAFs) in its progression. The researchers gathered data from various databases and used single-cell transcriptome analysis and ligand-receptor interaction analysis to identify CAF-related genes. They then developed an artificial neural network (ANN) model based on 12 prognostic CAF-related genes, creating a CAF activation score (CAS). Functional and immune analyses showed that high-CAS samples had more active cell crosstalk and immune activity. Mutational analysis revealed differentially mutated genes between high- and low-CAS samples. Clinical analysis resulted in a prognostic nomogram for HCC patients. A novel risk score (RS) based on CD4+ Tconv-related long non-coding RNAs (lncRNAs) was developed for HCC patients by (Zhu L. et al.). Their RS, consisting of six lncRNAs (AC012073.1, AL031985.3, LINC01060, MKLN1-AS, MSC-AS1, and TMCC1-AS1), demonstrated good predictive ability for overall survival (OS) in HCC patients and various clinical subgroups. Patients in the high-risk group exhibited an immune response phenotype characterized by high infiltration of macrophages and CAFs and low infiltration of natural killer (NK) cells. Furthermore, the low-risk group showed favorable responses to immune checkpoint inhibitors. Song et al. delved into the impact of DNA methylation regulators (DMRegs) on HCC. Their data clusters modifications based on DMRegs expression, genetics, and transcription in HCC samples. These alterations correlate with clinicopathological traits, prognosis, and immune cell infiltration patterns. The results introduces a DMRegs-related gene score (DMRegs_score) as a prognostic indicator, showing high scores are linked to poor outcomes. The DMRegs_score also shows promise in predicting drug sensitivity. Liu P. et al. also focused on HCC and the role of methylcytosine (m5C) regulators in predicting clinical responses to immunotherapy. Researchers analyzed data from 371 HCC patients and identified six differentially expressed genes (DEGs) to construct a prognostic risk model and two diagnostic models. The prognostic risk model effectively predicted patient outcomes, and the high-risk group showed a worse prognosis. Additionally, the high-m5C score group was predicted to be less responsive to immunotherapy but more sensitive to chemotherapy and potential targeted drugs. Overall, these insights offer potential for refining prognosis of HCC.

Furthermore, several strategies to help distinguish the potential efficiency of immune therapy were reported. Yuan G. et al. examined the significance of liver stiffness (LS) measured by shear wave elastography (SWE) in advanced HCC patients treated with PD-1 inhibitors and lenvatinib. A LS value of 19.53 kPa at baseline was identified as the optimal cutoff for predicting treatment efficacy. A nomogram combining baseline tumor LS and albumin-bilirubin grade was developed to predict treatment outcomes. High stiffness tumors were associated with metabolic pathways, while low stiffness tumors were related to DNA damage repair. Patients with high stiffness tumors had lower immune cell infiltration, suggesting potential drug candidates to enhance immunotherapy efficacy. Xu et al. developed a novel prognostic predictor for (PD-1 inhibitor therapy in HCC patients, independent of Child-Pugh grade. The study analyzed data from HCC patients who received PD-1 inhibitors and introduced a novel ALG grade based on serum ALP and GGT levels before treatment initiation. The results showed that patients with Child−Pugh grade A and ALG grade 3 at baseline had worse outcomes. From the gene level, chromosome 11q13 amplification (Amp11q13) was identified as a common variation in HCC patients. Yan et al. reported those with Amp11q13 tended to have higher levels of Des-γ-carboxy-prothrombin (DCP), more tumors, and were more likely to have portal vein tumor thrombosis (PVTT). In patients treated with PD-1 inhibitors, Amp11q13 was associated with a higher risk of progression, shorter progression-free survival (PFS), and a potential link to hyperprogressive disease (HPD). Liu C. et al. also identified predictive biomarkers for the effectiveness of combination therapy involving anti-angiogenic drugs and PD-1 antibodies in HCC patients. They collected data from 40 advanced HCC patients undergoing this combination therapy and found that high levels of CD3+CD4+CD279+ and CD3+CD8+CD45RO+CD62L+ T lymphocytes, as well as a high tumor mutational burden (TMB), were associated with a positive treatment response. Conversely, high levels of CD3+CD4+CD28+ T lymphocytes were linked to a poorer response. Specific gene mutations, such as TP53 and ARID1A, also correlated with non-response, while amplification mutations in 11q13-CCND1, FGF3, FGF4, and FGF19 were observed in a patient with hyperprogression. Interestingly, in a meta-analysis by Zongli Zhang et al., which focusing on HCC patients treated with immune checkpoint inhibitors (ICIs), the impact of antibiotic use on treatment outcomes was assessed. The analysis included six retrospective studies with 1056 patients, of which 33.33% received antibiotics. The results indicated that antibiotic use did not significantly affect OS or progression-free survival (PFS) in HCC patients treated with ICIs. Furthermore, antibiotics did not have a significant impact on objective response rate (ORR) or disease control rate (DCR). Therefore, their evidence suggests that antibiotics do not substantially alter the therapeutic efficacy of ICIs in HCC patients. A prognostic model was developed for unresectable HCC patients treated with a combination of ICIs and tyrosine kinase inhibitors (TKIs) by (Li X. et al.). Their model incorporates seven clinical parameters, including ECOG PS, TACE, EHM, PLR, ALT, AFP, and Child-Pugh score, which can help predict the efficacy of the combination regimen in unresectable HCC patients. Guo et al. established a prognostic model called the PIMET score for unresectable hepatocellular carcinoma (uHCC) patients receiving lenvatinib monotherapy or lenvatinib plus immune checkpoint inhibitors (ICI). The model includes metastasis and protein induced by vitamin K absence or antagonist-II (PIVKA-II) as risk factors. Patients were stratified into PIMET-low, PIMET-int, and PIMET-high groups. The PIMET score effectively predicted OS and treatment responses, distinguishing patients who benefit from the combination of lenvatinib and ICI. Taken together, stiffness tumors, ALG grade, clinical parameters (such as ECOG PS, TACE, EHM, PLR, ALT, AFP, and Child-Pugh score), Amp11q13, and mutations of certain genes (in 11q13-CCND1, FGF3, FGF4, and FGF19) could serve as potential predictive biomarkers for HCC patients undergoing immune therapy.

Several retrospective or prospective clinical studies were conducted regarding the treatment of HCC. For example, Xie et al. reported that combining ICIs with molecular targeted agents (MTAs) after lenvatinib progression in advanced hepatocellular carcinoma (aHCC) showed promising anticancer effects and safety. PFS and post-progression survival (PPS) were notably extended. No significant differences in efficacy were observed between ICI+Lenva and ICI+Others groups. Prolonged PPS was associated with Child–Pugh grade A, AFP < 400 IU/ml, and concomitant locoregional treatment. Adverse events were manageable. Wang K. et al. reviewed local ablative therapy in HCC. Radiofrequency ablation (RFA) is a primary treatment for early-stage HCC, but other techniques like microwave ablation, cryoablation, irreversible electroporation, and phototherapy are under investigation. Combining immunotherapy with ablation is a promising strategy, as ablative therapy can trigger local and systemic immune responses. Their review summarizes the current status of ablation and immunotherapy for HCC, explores the immune effects of ablation, and discusses combination strategies, including those involving biomedical materials. Combination therapies are attracting more and more attentions. Zeng et al.’s retrospective study aimed to compare the efficacy and safety of ICI plus bevacizumab (BEV) versus ICI plus receptor tyrosine kinase inhibitor (TKI) as first-line treatments for uHCC. The study included 94 patients and assessed PFS, OS, objective response rate (ORR), and disease control rate (DCR). While the median OS and PFS did not significantly differ between the two groups, the incidence of adverse events varied. Palmar-plantar erythrodysesthesia syndrome was more common in the ICI+TKI group, while upper gastrointestinal bleeding occurred primarily in the ICI+BEV group. Cai M. et al. compared the effectiveness and safety of two treatment approaches for advanced HCC. One group received transarterial chemoembolization combined with lenvatinib plus a PD-1 inhibitor (TACE-L-P), while the other received TACE combined with lenvatinib (TACE-L). Results showed that TACE-L-P led to longer overall survival (16.9 vs. 12.1 months), extended progression-free survival (7.3 vs. 4.0 months), and higher response rates. TACE-L-P was particularly beneficial for patients with extrahepatic metastasis or more than three tumors. Adverse events were similar between the two groups, therefore supporting TACE-L-P as a promising treatment for advanced HCC, especially in specific patient subgroups. Combining intensity-modulated radiotherapy (IMRT) with atezolizumab and bevacizumab (atezo/bev) in HCC patients with extrahepatic portal vein tumor thrombus (ePVTT) was also studied in a prospective multicenter research by (Wang et al.). The treatment demonstrated a promising objective response rate (76.6%) and a median overall survival of 9.8 months. There was no significant correlation found between tumor mutational burden (TMB) and treatment outcomes. The most common treatment-related adverse events were manageable, with no treatment-related deaths reported. Their approach appears to be a valuable option for HCC patients with ePVTT, although further research is needed for confirmation.

## 3 Bile duct cancer and gallbladder cancer

Cholangiocarcinoma represents a rare yet highly aggressive malignancy that emerges from the epithelial cells lining the bile ducts, responsible for transporting bile from the liver to the small intestine (11). This cancer is categorized into two primary forms: intrahepatic and extrahepatic cholangiocarcinoma, distinguished by their anatomical locations. Intrahepatic cholangiocarcinoma originates within the liver, while extrahepatic cholangiocarcinoma manifests outside the liver, typically within the bile ducts. Cholangiocarcinoma’s challenging clinical landscape arises from its elusive early symptoms, often leading to advanced-stage diagnoses with limited therapeutic options. However, contemporary research has been dedicated to unraveling its molecular underpinnings and identifying potential therapeutic targets, offering renewed optimism for the management of this intricate malignancy. Advances in precision medicine and immunotherapy have opened new avenues for developing more effective treatment modalities (12).

Li Y. et al. reviewed the function of bile, which directly contacts biliary tract tumors, contains complex components closely linked to BTC development. The bile components hold potential as biomarkers for BTCs. Furthermore, emerging evidence suggests that bile components play a role in regulating immune responses. Their review also explores the relationship between bile components and BTCs, their biomarker potential, and their immunoregulatory effects, highlighting their promising applications in BTC diagnosis and treatment.

As for the BTC treatment, Zhang Y. et al. reported that patients with advanced cholangiocarcinoma receiving PD-1 inhibitor combination therapy, experiencing immune-related adverse events (irAEs) was associated with better disease control, longer OS, and extended progression-free survival (PFS). These findings suggest that irAEs may serve as a positive predictor for treatment efficacy in this context. IrAEs were identified as independent prognostic factors for both OS and PFS, highlighting their potential as a valuable clinical indicator. Yang X. et al. found that for patients with unresectable intrahepatic cholangiocarcinoma (iCCA), DEB-TACE+ICIs demonstrated superior outcomes compared to gemcitabine+cisplatin chemotherapy. DEB-TACE+ICIs led to higher objective response rates, extended PFS, and prolonged OS. Independent risk factors for worse PFS and OS included chemotherapy, tumor size >5cm, and multiple tumors. The incidence of treatment-related adverse events was comparable between groups. Therefore, they concluded that DEB-TACE+ICIs was a more effective and well-tolerated treatment approach for unresectable iCCA patients compared to chemotherapy. Zhang W. et al. showed that combining lenvatinib, pembrolizumab, and GP chemotherapy is promising for a patient with initially unresectable ICC. After six cycles of treatment, the tumors shrank, and tumor marker levels normalized. The patient underwent successful liver resection with no signs of recurrence or metastasis after 15 months. Jiang et al. conducted a meta-analysis involving advanced BTC patients, the effectiveness and safety of anti-PD1/PDL1 therapy were evaluated. The combination of anti-PD1/PDL1 with anti-CTLA4 and chemotherapy demonstrated the best outcomes, with a median PFS of 12.4 months, median OS of 16.0 months, a 45.1% ORR, and a 95.0% DCR. Anti-PD1/PDL1 monotherapy had the lowest efficacy but a safer profile. Overall, anti-PD1/PDL1 therapies showed promising efficacy and could be considered an alternative for aBTC treatment, despite some associated toxicities, particularly in the first-line setting. Another meta-analysis by Xian et al. aimed to assess the prognostic value of PD-L1 expression in ICC patients. Ten trials involving 1944 cases were analyzed. The low-PD-L1 group had significantly better OS, recurrence-free survival (RFS), and time to relapse compared to the high-PD-L1 group. Additionally, high PD1 levels were associated with poorer OS and RFS. Multivariate analysis confirmed PD-L1 and PD1 as independent predictors for OS and RFS. These findings suggest that PD-L1/PD1 expression can serve as valuable prognostic and predictive biomarkers in ICC and potential therapeutic targets. However, the adverse effect should not be ignored. For example, Zhu S. et al. reported a 48-year-old male ICC case with non-bacterial cystitis as an immune-related adverse event (irAE) following treatment with PD-1 and PD-L1 antibodies. In that case, psoriasis worsened, and urinary discomfort recurred, leading to treatment discontinuation and surgery. After chemotherapy with atezolizumab, urinary discomfort reappeared. Urine cultures showed no bacteria, and cystoscopy biopsy indicated non-bacterial bladder inflammation. This highlights a rare case of immunotherapy-induced non-bacterial urinary tract inflammation. On the other hand, Wang Y. et al. reviewed the antiangiogenic therapy in controlling BTC progression. Understanding the molecular basis of angiogenesis in BTCs is crucial for treatment strategies and patient selection. This review summarizes recent advances in antiangiogenic approaches for BTCs, emphasizing molecular mechanisms and clinical trial outcomes. The potential future of antiangiogenic therapy in BTCs is also discussed, offering insights into this challenging malignancy’s management.

Gallbladder cancer is a rare cancer which are mostly reported as case reports (13, 14). Wu et al. reported that, in the context of uncertain ICI benefits in gallbladder cancer, a 45-year-old female with multiple abdominal lymph node metastases received camrelizumab combined with paclitaxel and gemcitabine (AG) to shrink the tumor before surgery. Postoperatively, her quality of life improved. Camrelizumab + AG presents a potential treatment for gallbladder cancer with such metastases but requires confirmation through clinical trials. Another case by Zhang Y. et al. reports successful treatment of advanced gallbladder cancer with high PD-L1 expression or high tumor mutation burden (TMB-H) using a combination of tislelizumab and S-1. Most patients experienced effective tumor control, while one had immune-related pneumonia (irP) resolved with therapy and surgery. Despite tumor control resuming after surgery, recurrent irP led to discontinuation and tumor progression. The findings suggest that combining anti-PD-1 antibodies with S-1 is a safe and effective treatment for GBC, particularly for biomarker-positive cases, offering a novel approach to advanced GBC therapy.

## 4 Pancreatic cancers

Pancreatic cancer is a formidable and often fatal malignancy that originates in the pancreas, a crucial organ with both endocrine and exocrine functions. Pancreatic ductal adenocarcinoma (PDAC) represents the most prevalent histological subtype, responsible for the majority of pancreatic cancer cases. Characterized by rapid progression, early metastasis, and resistance to conventional therapies, pancreatic cancer has long posed a significant clinical challenge (15). Recent research efforts have expanded our comprehension of the intricate molecular alterations and the complex tumor microenvironment driving its pathogenesis (16). Advances in early detection methods, targeted therapies, and immunotherapies have emerged as promising avenues for combating pancreatic cancer, offering renewed hope for improved patient outcomes (17).

Zhang Y. et al. explored the clinical and functional significance of F-box only proteins (FBXOs) in PDAC. They identified six FBXOs (FBXO1, FBXO20, FBXO22, FBXO28, FBXO32, and FBXO45) that were significantly upregulated in PDAC tissues, correlating with adverse patient prognosis and clinicopathological features. Promoter methylation influenced FBXO expression, and genetic alterations and mutations in these FBXOs affected patient outcomes. Additionally, the study revealed associations between FBXOs and immune infiltrations, including B cells, T cells, NK cells, macrophages, and dendritic cells, suggesting their role in the immune response. Functional analysis implicated these FBXOs in various signaling pathways, making them potential diagnostic and therapeutic targets for PDAC. Similarly, Sijde et al. investigated the role of circulating cytokines in predicting treatment response and overall survival in PDAC patients undergoing FOLFIRINOX chemotherapy. High levels of IL-1RA after one cycle of chemotherapy are associated with reduced tumor progression during treatment. Additionally, serum concentrations of IL-7, IL-18, and MIP-1β after one cycle of FOLFIRINOX correlate with overall survival. These findings suggest that specific cytokines and immune cells play crucial roles in chemotherapy response and PDAC progression, potentially paving the way for cytokine-based treatments in the future. Eckhoff et al. showed that in patients with intraductal papillary mucinous neoplasm (IPMN) and PDAC, the activity of peripheral blood monocytes, specifically TNF expression upon R848 stimulation, inversely correlates with disease progression. Patients with low-grade IPMN and stage 1 PDAC exhibit higher TNF expression compared to those with high-grade IPMN and stage 2/3 PDAC, indicating innate immune reprogramming as IPMNs progress to invasive cancer. Additionally, sera from IPMN and PDAC patients contribute to the suppression of TNF induction in healthy donor monocytes, suggesting the involvement of soluble mediators in this process. Yuan H. et al. focused on 5-methylcytosine (m5C) modification in long non-coding RNAs (lncRNAs) and its implications in PDAC. Utilizing clinical data and genetic transcriptome information from the TCGA database, the researchers conducted bioinformatic analyses to establish an m5C-related lncRNA prognostic risk model for PDAC patients. This model effectively distinguished between PDAC and normal tissues and accurately predicted survival outcomes for PDAC patients. Zhuang et al. aimed to develop a prognostic classifier to assess hypoxia status and related molecular characteristics in PDAC. They classified PDAC into three clusters based on 16 known hypoxia-related genes and identified nine differentially expressed genes to construct an HIF-1 score system. This system effectively predicted patient survival outcomes and demonstrated superior predictive ability compared to previous hypoxia signatures. Furthermore, the study explored the oncogenic pathways and immune cell infiltration status associated with different HIF-1 scores, highlighting the potential for combination treatment strategies for highly hypoxic and immunosuppressive PDAC tumors. Su et al. also aimed to develop an immune-related gene prognostic risk index (IRGPRI) for pancreatic adenocarcinoma (PAAD) and explore its implications. Using the TCGA and GEO datasets, 16 immune-related hub genes were identified to construct the IRGPRI. Low IRGPRI was associated with favorable outcomes, immune-related pathways, and higher benefit from ICIs. High IRGPRI correlated with cancer-related pathways, less favorable outcomes, and lower benefit from ICIs. Chen X. et al. believe that the extracellular matrix (ECM) is crucial in the tumor microenvironment, affecting cancer cell behavior. Therefore their study explored ECM-related genes (ECMGs) in PAAD and pan-cancer contexts. Seven ECMGs were identified as PAAD hub genes, associated with tumor stage and prognosis. ECM-based subtypes showed distinct features in oncogene/tumor suppressor gene expression, the immune environment, and chemotherapy sensitivity. A prognostic panel, combining ECM-related mRNAs and lncRNAs, was developed and validated for accurate PAAD prognosis prediction.

## 5 Pan-cancer

Pan-cancer analyses, especially in-depth reviews, are critical for a comprehensive understanding of certain tumor-related biomarkers (18, 19). For example, Zhao et al. reviewed the Structural Maintenance of Chromosome 4 (SMC4), a member of the ATPase family involved in various cellular processes such as chromosome organization, DNA repair, and genome transcription. This review delves into the multifaceted functions of SMC4, including its role in cell division, RNA splicing, DNA metabolism, and the immune response. The focus is on its relevance in cancer, where high SMC4 expression is consistently associated with poorer overall survival. By analyzing data from various sources, this review suggests that SMC4 could serve as a valuable prognostic marker and potential therapeutic target in cancer. MicroRNAs (miRNAs) have gained significant attention in cancer research due to their pivotal role in tumorigenesis. Lu et al. reviewed the role of miR-608 as a tumor suppressor, which had been found to be downregulated, particularly in solid tumors. Extensive *in vivo* and *in vitro* experiments have verified its tumor-inhibiting properties. MiR-608 exerts its influence on various biological processes, including cell proliferation, invasion, migration, and apoptosis, by targeting transmembrane proteins and modulating key signaling pathways. This review summarizes miR-608’s expression profile, biological functions, and underlying mechanisms, underscoring its potential as a diagnostic, prognostic biomarker, and therapeutic target in cancer. Another review by Wang J. et al. focused on immunotherapy, especially ICI, which is entering a new era of precision medicine. Clinical benefits of ICI in digestive system cancers are limited, and it often comes with side effects and high costs. To address this, the development of biomarkers for predicting immunotherapy effectiveness is crucial. They reviewed various potential biomarkers, including microsatellite mismatch repair, tumor mutation burden, specific mutated genes or pathways, PD-L1 expression, immune-related adverse reactions, blood biomarkers, and patient-related factors in predicting immunotherapy efficacy against digestive system cancers. The establishment of dynamic personalized prediction models based on multiple biomarkers holds promise for future research in this field.

Ti et al. examined squamous cell carcinomas (SCCs) from different sites to investigate the relationship between tumor mutation burden (TMB) and prognosis and immune cell infiltration in SCCs. TMB had varying effects on prognosis in different SCCs; it was associated with better prognosis in lung and cervical SCCs but worse prognosis in head and neck and esophageal SCCs. Older age, smoking history, earlier stage, and no lymphatic invasion were linked to higher TMB. Immune-related genes and immune cell proportions also varied between high and low TMB groups, thus provides insights for immunotherapy biomarkers in SCCs. In HPB malignancies, various T cell types with immune checkpoint receptors exist. Wan et al. reported that HCC shows a favorable presence of tissue resident memory T cells that respond well to immune checkpoint therapies. In contrast, pancreatic ductal adenocarcinoma (PDA) has terminally differentiated T cells with less potential for activation, regardless of chemotherapy. Combining checkpoint therapies may benefit HCC, while alternative approaches are needed for CCA and PDA. Shen et al. conducted a phase II clinical trial for advanced solid tumors resistant to standard treatments, in which a combination therapy was evaluated. Patients received targeted radiotherapy followed by liposomal irinotecan, camrelizumab, and anti-angiogenic drugs. Among 52 evaluated patients, there was a 34.6% objective response rate and an 82.7% disease control rate. Median progression-free survival was 5.3 months, and overall survival was not reached during the study period. Taken together, although treatment-related adverse events were common, the combination therapy showed promising anti-tumor activity and good tolerability in diverse advanced solid tumors.

As genomics technology continues to progress, it opens up new avenues for medical research and patient care. The analysis of gene expression profiles, coupled with the integration of patients’ clinical information, medical imaging, and laboratory data, holds the potential to revolutionize the field. Emerging bioinformatics techniques, such as machine learning algorithms, promise to play a pivotal role in this transformation, enabling the creation of predictive models that are both highly accurate and robust. These models, in turn, will serve as invaluable tools in guiding medical decision-making. This wave of research endeavors is poised to focus on the development of personalized therapeutic drugs and treatment strategies, which aim to enhance treatment efficacy while minimizing unnecessary risks to patients. A deeper exploration of the intricate mechanisms underlying immunotherapies and chemotherapies will pave the way for the identification of novel therapeutic targets and innovative combination treatment approaches. Collectively, these advances are steering the medical field towards a future characterized by personalized treatment and intelligent medical care. This progressive trajectory promises to provide patients with increasingly precise and effective healthcare services, ultimately improving their overall well-being.

## Author contributions

YQ: Writing – original draft. WJ: Writing – review & editing. HL: Writing – review & editing.
